# NKG2D as a Cell Surface Marker on γδ-T Cells for Predicting Pregnancy Outcomes in Patients With Unexplained Repeated Implantation Failure

**DOI:** 10.3389/fimmu.2021.631077

**Published:** 2021-03-10

**Authors:** Chunyu Huang, Zheng Xiang, Yongnu Zhang, Yuye Li, Jian Xu, Hongzhan Zhang, Yong Zeng, Wenwei Tu

**Affiliations:** ^1^Department of Paediatrics and Adolescent Medicine, Li Ka Shing Faculty of Medicine, University of Hong Kong, Hong Kong, China; ^2^Shenzhen Key Laboratory of Reproductive Immunology for Peri-implantation, Shenzhen Zhongshan Institute for Reproduction and Genetics, Fertility Center, Shenzhen Zhongshan Urology Hospital, Shenzhen, China

**Keywords:** γδ-T cells, NKG2D, unexplained repeated implantation failure, activation and inhibitory receptors, receiver operating characteristic curve

## Abstract

Maternal immune tolerance to semi-allogeneic fetus is essential for a successful implantation and pregnancy. Growing evidence indicated that low cytotoxic activity of γδ-T cells, which is mediated by activation and inhibitory receptors, is important for establishment of maternal immune tolerant microenvironment. However, the correlation between receptors on peripheral blood γδ-T cells, such as NKG2D, CD158a, and CD158b, and pregnancy outcome in patients with unexplained repeated implantation failure (uRIF) remains unclear. In this study, the association between the expression level of these receptors and pregnancy outcome in patients with uRIF was investigated. Thirty-eight women with uRIF were enrolled and divided into two groups: successful group and failed group, according to the pregnancy outcome on different gestational periods. The percentage of NKG2D^+^ γδ-T cells in lymphocytes was significantly higher in uRIF patients who had failed clinical pregnancy in subsequent cycle, compared with those who had successful clinical pregnancy. However, there were no differences about the frequencies of CD158a^+^ and CD158b^+^ γδ-T cells between the successful and failed groups. The receiver operating characteristic curve exhibited that the optimal cut-off value of NKG2D^+^ γδ-T cells was 3.24%, with 92.3% sensitivity and 66.7% specificity in predicting clinical pregnancy failure in uRIF patients. The patients with uRIF were further divided into two groups, group 1 (NKG2D^+^ γδ-T cells <3.24%) and group 2 (NKG2D^+^ γδ-T cells ≥3.24%), based on the cut-off value. The live birth rate of patients in the group 1 and group 2 were 61.5 and 28.0%, respectively. Kaplan-Meier survival curve further suggested that the frequency of NKG2D^+^ γδ-T cells in lymphocytes negatively correlated with live birth rate in patients with uRIF. In conclusion, our study demonstrated that the frequency of peripheral blood NKG2D^+^ γδ-T cells among lymphocytes is a potential predictor for pregnancy outcome in uRIF patients.

## Introduction

The current clinical definition of infertility is the state of a diminished capacity to conceive after 12 months of regular sexual intercourse. The prevalence of infertility is ~10% among men and 13% among women (1). Although assisted reproductive technologies helped numerous infertile couples in achieving a successful pregnancy, 10–15% of the infertile couples who were subjected to assisted reproductive technology treatment experienced failure to conceive after multiple *in vitro* fertilization-embryo transfer (IVF-ET) cycles (2). These patients are defined as repeated implantation failure (RIF) patients. The etiology of RIF is complex, mainly including genetic abnormalities, endocrine disorders, abnormal anatomy of the genital tract, presence of infection and/or some autoimmunity diseases, and thrombophilia conditions (3). After ruling out the RIF patients with above common etiology, there are still some patients with unknown etiology. These RIF patients are defined as unexplained RIF (uRIF) patients.

The maternal-fetal interface is an indispensable aspect to establish and maintain the successful pregnancy. On one hand the extra villous trophoblasts invade the decidua and directly interact with decidual immune cells, on the other hand the syncytiotrophoblast provides a connection with the uterus wall to allow the contact of the maternal circulation with the fetus for transport of nutrients (4). Therefore, an appropriate recognition of the semi-allogeneic fetus by the maternal immune system is required for establishing an immune tolerant microenvironment which allows a successful implantation and pregnancy (5). In this context, γδ-T cells, a group of unconventional T cells that express the T cell receptor containing a γ and δ chain, display an important role due to their early and rapid response against cellular stress and infections in an major histocompatibility complex (MHC)-independent manner (6). Although γδ-T cells represent a minor population of T lymphocytes (1–10%) (7, 8), they exhibit multiple immunological functions, such as anti-viral infection, inflammation regulation, tissue homeostasis, and even in maintaining successful pregnancy (9–13). In the early 1990s, some studies suggested that peripheral γδ-T cells play an important role in decreasing natural killer activity through secreting anti-inflammatory cytokines during the whole pregnancy process (14, 15). In addition, the low cytotoxic activity of γδ-T cells is essential during a normal pregnancy (16). The γδ-T cell subsets with potent cytotoxic activity were associated with unexplained recurrent miscarriage (17). However, the correlation between γδ-T cells and uRIF still remains unknown.

Cytotoxic activity of γδ-T cells is mainly mediated by releasing soluble cytotoxic granules, such as perforin, granzyme and granulysin (18, 19). Most importantly, the degranulation process is regulated by the balance between activation and inhibitory receptors (20). For instance, NKG2D is an activation receptor expressed on most human NK cells and γδ-T cells. The interaction between NKG2D and its ligands can modulate the cytotoxic capacity of γδ-T cells (21–23). Indeed, the activation of the NKG2D pathway induces the secretion of cytotoxic granules, interferon-γ (IFN-γ) and tumor necrosis factor-α (TNF-α), finally promoting the cytotoxicity of γδ-T cells against infected cells or tumor cells (24, 25). In contrast, regulatory receptors for self-MHC class I molecules, particularly killer cell immunoglobulin-like receptors (KIRs), negatively regulate γδ-T cells activation (26). KIR with two Ig domains (KIR2D), such as KIR2DL1 (CD158a) and KIR2DL2/L3 (CD158b), which can bind to the human leukocyte antigen (HLA)-C. This interaction activates the downstream immunoreceptor tyrosine-based inhibitory motifs to inhibit the signal activation (27). Previous studies suggested that CD158a^+^/CD158b^+^ γδ-T cells were less cytotoxic than CD158a^−^/CD158b^−^ γδ-T cells (28, 29). However, up to now, little is known regarding the relationship between expression pattern of NKG2D, CD158a and CD158b on γδ-T cells and pregnancy outcome in uRIF patients.

In this study, we demonstrated that high frequency of NKG2D^+^ γδ-T cells in lymphocytes was inversely correlated with pregnancy outcomes in patients with uRIF. Our data suggest that the frequency of NKG2D^+^ γδ-T cells in lymphocytes could be used to predict pregnancy outcome in uRIF patients.

## Materials and Methods

### Study Population

**Figure 1:**
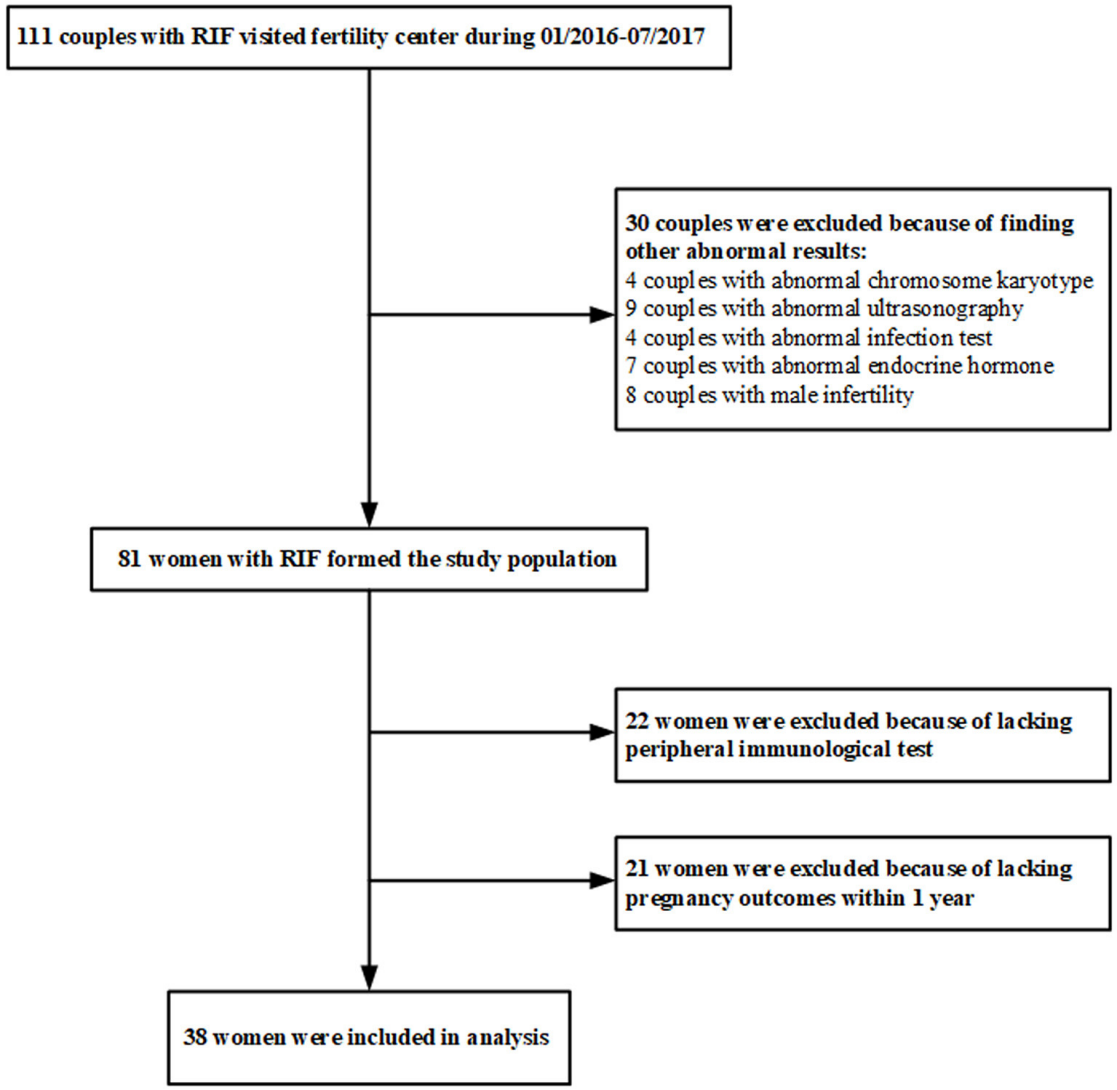
Flowchart listing the inclusion and exclusion criteria to select patients in this study.

Female infertile patients were selected from infertile couples who went to the Fertility Center of Shenzhen Zhongshan Urology Hospital, China, from January 2016 to July 2017. The inclusion criteria include: (1) age of 18–40 years; (2) met the diagnostic criteria of RIF: three or more IVF failures after the transfer of at least one high-quality embryo per cycle. The potential conventional factors causing RIF were not taken into consideration at the time of selection. The exclusion criteria include: (1) abnormal chromosome karyotype; (2) abnormal ultrasonography and hysterosalpingogram/hysteroscopy result; (3) infection of hepatitis B virus, hepatitis C virus, human immunodeficiency virus, toxoplasma, rubella virus, cytomegalovirus, or herpes virus; (4) abnormal basal endocrine hormone level, such as follicle-stimulating hormone, estradiol, luteinizing hormone, prolactin, or testosterone; (5) male infertility. The peripheral blood of the patients enrolled in this study was collected to perform immunological assays, and then these patients received an empirical therapy represented by the endometrium scratching operation. Women who did not provide the blood sample for immunological assays were excluded. To match the end-points of the study, women who were not subjected to embryo transfer operation within 1 year after the immunological tests were also excluded. A total of 111 couples met the diagnostic criteria of RIF and were enrolled in the study (Figure 1). After excluding 30 subjects with conventional factors, a total of 81 uRIF women comprised the study population. There were 22 subjects who failed immunologic testing and 21 women who did not undergo embryo transfer within 1 year after immunologic testing, resulting in a total of 38 uRIF women who participated in the statistical analysis.

To assess the power value of the receptors on the γδ-T cell in predicting the outcome of pregnancy failure in patients with uRIF on different gestational periods, implantation, clinical pregnancy, ongoing pregnancy, and live birth were followed up. Potential successful implantation was monitored at ~14 days after embryo transfer and was defined as the presence of seropositive-human chorionic gonadotropin. Otherwise, implantation failure was defined. A successful clinical pregnancy was defined as the presence of a gestational sac as shown by ultrasound 4–5 weeks after embryo transfer. If absent, a failed clinical pregnancy was defined. Successful sustained pregnancy was defined as the presence of a live fetus of ~12 weeks gestational age. Otherwise, it was defined as a failure of the ongoing pregnancy. Successful live birth was defined as the delivery of a live fetus by the mother at full term gestation, otherwise, it was defined as failure of live birth.

This study was performed in accordance with the Declaration of Helsinki, and the protocol was approved by the Ethics Committee of Shenzhen Zhongshan Urology Hospital. All participants gave their informed consent for the inclusion in this study before their participation.

### Flow Cytometry

Immunological phenotypes of γδ-T cells and their subpopulations were determined by flow cytometry. The following fluorophore-conjugated mouse monoclonal antibodies anti-human antibodies were used: PerCP-conjugated anti-CD3 (347344; BD Pharmingen, USA), PE-cy7-conjugated anti- TCRγδ (331222; Biolegend, USA), FITC-conjugated anti-NKG2D (11-5878; eBioscience, USA), FITC-conjugated anti-CD158a (556062; BD Pharmingen, USA), PE-conjugated anti-CD158b (559785; BD Pharmingen, USA).

### Phenotypic Characteristics of γδ-T Cells

**Figure 2:**
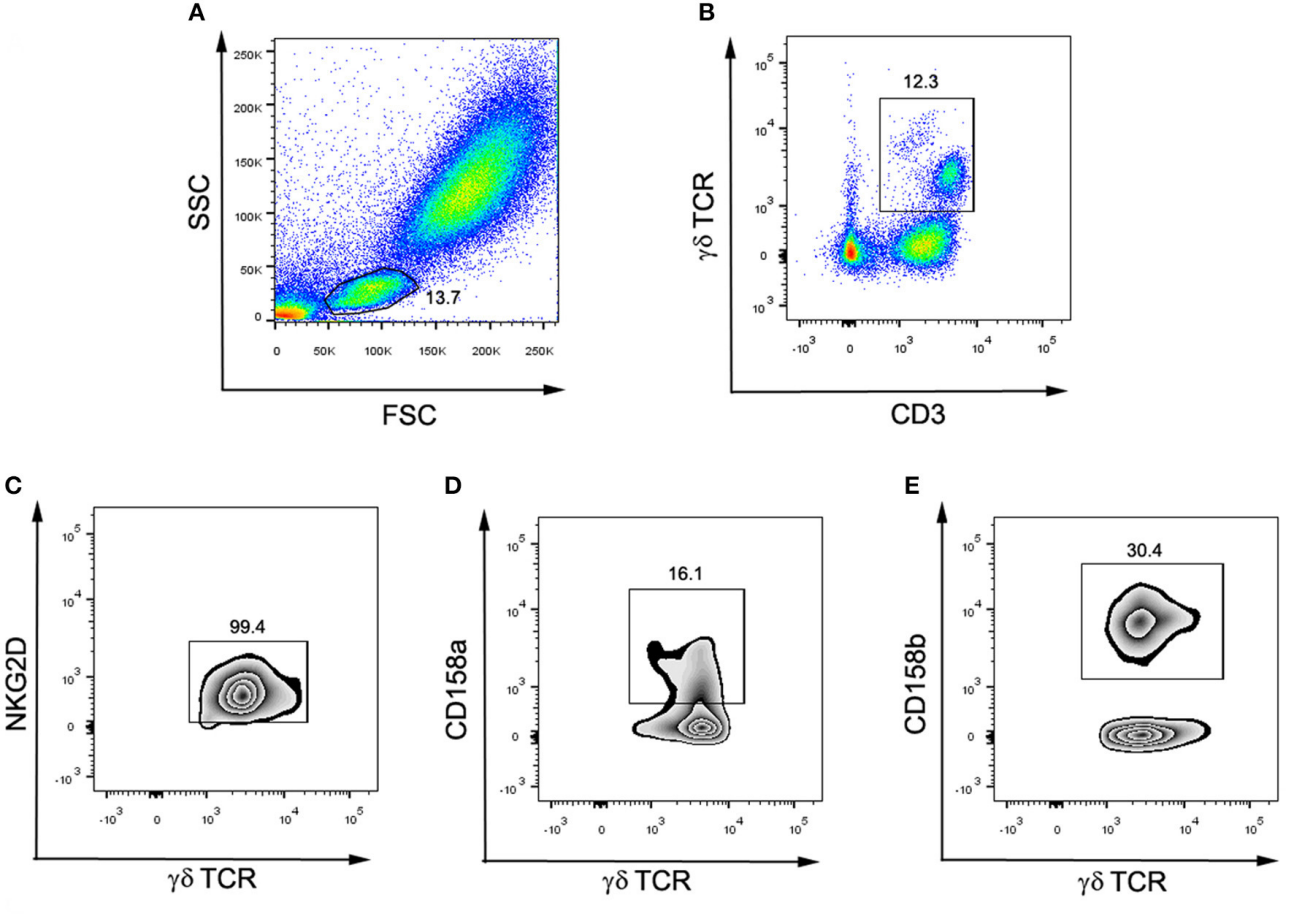
Flow cytometry gating strategy for surface markers and γδ-T cell subsets. **(A)** The lymphocytes were gated by FSC and SSC. **(B)** CD3^+^ γδ TCR^+^ cells were defined as γδ-T cells. **(C)** Among γδ-T cells, NKG2D^+^γδ TCR^+^ were defined as NKG2D^+^ γδ-T cells. **(D)** Among γδ-T cells, CD158a^+^γδ TCR^+^ were defined as CD158a^+^ γδ-T cells. **(E)** Among γδ-T cells, CD158b^+^γδ TCR^+^ were defined as CD158b^+^ γδ-T cells.

To determine the surface expression of activation markers on the surface of γδ-T cells, the heparinized whole blood (100 μL) was collected during the mid-luteal phase of the menstrual cycle and was stained with anti-CD3 (5 μL), anti-TCRγδ (5 μL), and anti-NKG2D (10 μL) antibodies. To determine the surface expression of inhibitory markers on γδ-T cells, the heparinized whole blood (100 μL) was incubated for 15 min with antibodies against CD3 (5 μL), γδ TCR (5 μL), CD158a (10 μL), and CD158b (10 μL). After 15 min of incubation in the dark, the whole blood was lysed using 1×FACS lysis solution, and the cells were subsequently washed three times with PBS. Finally, cells were analyzed with a BD FACSCanto II flow cytometer (BD, USA). According to the gating strategy (Figure 2), the lymphocytes were gated by FSC and SSC first. Then CD3^+^ TCRγδ^+^ cells were defined as γδ-T cells. In the population of γδ-T cells, TCRγδ^+^NKG2D^+^ cells, TCRγδ^+^CD158a^+^ cells, and TCRγδ^+^CD158b^+^ cells were defined as NKG2D^+^ γδ-T cells, CD158a^+^ γδ-T cells and CD158b^+^ γδ-T cells, respectively. The percentage of receptor-positive γδ-T cells in total γδ-T cells, CD3^+^ T cells, and overall lymphocytes was respectively calculated.

### Statistical Analysis

Statistical analysis was performed using SPSS 20.0 (IBM, USA). Kolmogorov-Smimov test was used to determine the normal distribution of the data. Data were presented as mean ± SD when normally distributed, or as median with quartiles when not normally distributed. Inter-group differences in immune markers were examined using the independent *t*-test when the data were normally distributed, or using the Mann-Whitney *U*-test when they were not normally distributed. The categorical variables were analyzed by chi-square test or Fisher's exact test. Receiver operating characteristic (ROC) curve and the area under the curve (AUC) were used to establish the power value of the immune markers in predicting the pregnancy outcome in patients with uRIF. The overall live birth rate and gestational age of the patients with uRIF were estimated by the Kaplan-Meier analysis. The time to event was referred to the length in weeks from the date of the embryo transfer to the date of the patient undergoing pregnancy failure, or to the date of the end point of the pregnancy in patients who gave live birth, or to the date of loss to follow-up (censored). A two-tailed *P*-value of < 0.05 was considered statistically significant.

## Results

### Clinical Characteristics for uRIF Patients Categorized by Pregnancy Outcomes

To investigate the relationship between basic clinical characteristics and pregnancy outcomes, basic clinical characteristics were compared between uRIF patients with successful pregnancy outcome and patients with failed pregnancy outcome on different gestational periods. As shown in Table 1, regardless of the gestational period, there was no evident difference in the clinical characteristics, such as age, body mass index, number of previous failed pregnancy, content of estradiol, progesterone, follicle-stimulating hormone, luteinizing hormone, prolactin, and testosterone (all *P* > 0.05), between the successful group and failed group. Other factors which potentially affect the pregnancy outcome during IVF-ET treatment process, such as percentage of blastocyst in the transferred embryos, number of transferred embryos, number of transferred high-quality embryos were also comparable between the successful group and failed group.

**Table 1:** Clinical characteristics of uRIF patients categorized according to the pregnancy outcome.

**Factors**	**Implantation**		***P***	**Clinical pregnancy**		***P***	**Ongoing pregnancy**		***P***	**Live birth**		***P***
	**Successful (*n* = 23)**	**Failed (*n* = 15)**		**Successful (*n* = 20)**	**Failed (*n* = 18)**		**Successful (*n* = 16)**	**Failed (*n* = 22)**		**Successful (*n* = 15)**	**Failed (*n* = 23)**	
Age (years)	34.7 ± 4.1	36.3 ± 3.5	0.221	34.8 ± 4.4	35.9 ± 3.4	0.380	34.6 ± 4.6	35.8 ± 3.3	0.338	34.5 ± 4.8	35.8 ± 3.3	0.346
BMI (kg/m^2^)	21.3 ± 2.3	21.7 ± 2.0	0.502	21.1 ± 2.4	21.7 ± 1.9	0.435	20.9 ± 2.3	21.8 ± 2.0	0.206	20.9 ± 2.4	21.8 ± 1.9	0.233
No. of previous pregnancy failure	4.9 ± 2.0	4.1 ± 2.4	0.244	4.9 ± 2.0	4.2 ± 2.4	0.342	5.3 ± 2.0	4.1 ± 2.2	0.104	5.2 ± 2.0	4.2 ± 2.2	0.156
E2 (pg/mL)	40.5 ± 11.9	40.3 ± 20.4	0.963	42.3 ± 11.4	38.3 ± 19.3	0.434	42.7 ± 12.8	38.8 ± 17.4	0.449	42.9 ± 13.2	38.9 ± 17.0	0.445
P (ng/mL)	0.3 ± 0.1	0.3 ± 0.2	0.616	0.3 ± 0.1	0.3 ± 0.2	0.513	0.3 ± 0.1	0.3 ± 0.2	0.491	0.3 ± 0.1	0.3 ± 0.2	0.487
FSH (mIU/mL)	6.8 ± 1.7	6.8 ± 2.0	0.953	6.5 ± 1.2	7.0 ± 2.3	0.403	6.4 ± 1.3	7.0 ± 2.1	0.360	6.4 ± 1.4	7.0 ± 2.0	0.355
LH (mIU/mL)	5.1 ± 1.8	4.0 ± 1.5	0.050	4.9 ± 1.6	4.4 ± 1.9	0.373	4.7 ± 1.5	4.6 ± 1.9	0.912	4.7 ± 1.5	4.6 ± 1.9	0.911
PRL (ng/mL)	19.5 ± 5.2	18.1 ± 5.5	0.408	19.8 ± 5.2	18.1 ± 5.4	0.331	19.7 ± 5.8	18.4 ± 5.0	0.470	19.7 ± 6.0	18.4 ± 4.9	0.465
T (ng/mL)	0.6 ± 0.3	1.1 ± 2.1	0.380	0.6 ± 0.3	1.0 ± 2.0	0.430	0.6 ± 0.3	0.9 ± 1.8	0.513	0.6 ± 0.3	0.9 ± 1.7	0.509
Blastocyst/transferred embryos	52.2%	33.3%	0.326	50.0%	38.9%	0.532	56.2%	36.4%	0.324	60.0%	34.8%	0.185
No. of transferred embryos	1.9 ± 0.5	2.2 ± 0.7	0.106	1.9 ± 0.6	2.2 ± 0.6	0.114	1.8 ± 0.7	2.1 ± 0.6	0.110	1.9 ± 0.6	2.1 ± 0.6	0.287
No. of high-quality embryos	1.4 ± 0.8	1.6 ± 0.9	0.445	1.4 ± 0.8	1.6 ± 0.9	0.575	1.3 ± 0.9	1.6 ± 0.8	0.343	1.4 ± 0.9	1.6 ± 0.8	0.445

*BMI, body mass index; E2, estradiol; P, progesterone; FSH, follicle-stimulating hormone; LH, luteinizing hormone; PRL, prolactin; T, testosterone*.

### Expression of NKG2D on γδ-T cells Were Increased in uRIF Patients With Clinical Pregnancy Failure

To investigate the relationship between surface receptors on γδ-T cells and pregnancy outcome in patients with uRIF, the percentage of NKG2D^+^, CD158a^+^ or CD158b^+^ γδ-T cells in γδ-T cells, CD3^+^ T cells or overall lymphocytes were compared between the successful group and failed group, respectively. As shown in Table 2, there were no significant differences in the percentages of NKG2D^+^ γδ-T cells, CD158a^+^ γδ-T cells and CD158b^+^ γδ-T cells in total γδ-T cells between the successful group and failed group. The percentages of total γδ-T cells, NKG2D^+^ γδ-T cells, CD158a^+^ γδ-T cells and CD158b^+^ γδ-T cells in CD3^+^ T cells were similar between the successful group and failed group. In addition, no significant differences were found on the percentages of CD158a^+^ γδ-T cells and CD158b^+^ γδ-T cells in the overall lymphocytes between the successful group and failed group. However, the percentages of CD3^+^ T cells [(62.5 ± 6.9)% vs. (59.5 ± 24.0)%, *P* = 0.016], γδ-T cells [(8.0 ± 6.7)% vs. (4.5 ± 2.1)%, *P* = 0.047], and NKG2D^+^ γδ-T cells [(7.6 ± 6.5)% vs. (3.8 ± 2.1)%, *P* =0.025] in lymphocytes were significantly increased in uRIF patients with clinical pregnancy failure, when compared to that in uRIF patients with successful clinical pregnancy. Taken together, our results suggested that the increase of NKG2D^+^ γδ-T cell frequency in lymphocytes might be associated with clinical pregnancy failure in uRIF patients.

**Table 2:** Comparison of frequency of γδ-T subsets in uRIF patients with successful pregnancy and those patients with pregnancy failure.

**Cell populations**	**Implantation**		***P***	**Clinical pregnancy**		***P***	**Ongoing pregnancy**		***P***	**Live birth**		***P***
	**Successful (*n* = 23)**	**Failed (*n* = 15)**		**Successful (*n* = 20)**	**Failed (*n* = 18)**		**Successful (*n* = 16)**	**Failed (*n* = 22)**		**Successful (*n* = 15)**	**Failed (*n* = 23)**	
**In** **γδ-T cells (%)**												
NKG2D^+^ γδ-T cells	79.2 ± 25.0	91.1 ± 5.2	0.078	81.3 ± 21.0	86.8 ± 20.0	0.077	82.9 ± 21.0	84.6 ± 20.4	0.438	82.7 ± 21.7	84.7 ± 20.0	0.595
CD158a^+^ γδ-T cells	13.1 ± 10.6	11.9 ± 11.2	0.734	13.4 ± 11.2	11.8 ± 10.5	0.660	11.7 ± 9.2	13.3 ± 11.9	0.660	12.2 ± 9.3	12.9 ± 11.7	0.836
CD158b^+^ γδ-T cells	18.5 ± 16.4	17.0 ± 13.6	0.778	18.5 ± 17.3	17.2 ± 13.0	0.789	22.1 ± 17.6	14.8 ± 12.7	0.149	23.3 ± 17.5	14.3 ± 12.6	0.075
**In CD3**^**+**^ **T cells (%)**												
γδ-T cells	8.4 ± 3.7	13.0 ± 10.5	0.120	8.2 ± 3.8	12.5 ± 9.6	0.090	8.4 ± 4.2	11.5 ± 8.9	0.216	8.8 ± 4.1	11.1 ± 8.9	0.352
NKG2D^+^ γδ-T cells	8.0 ± 3.8	12.4 ± 10.4	0.140	7.7 ± 4.0	12.0 ± 9.5	0.093	8.1 ± 4.3	10.9 ± 8.9	0.254	8.5 ± 4.2	10.5 ± 8.9	0.405
CD158a^+^ γδ-T cells	1.3 ± 1.1	2.1 ± 2.9	0.378	1.4 ± 1.1	1.9 ± 2.7	0.418	1.3 ± 1.1	1.8 ± 2.5	0.432	1.4 ± 1.1	1.8 ± 2.4	0.584
CD158b^+^ γδ-T cells	1.6 ± 1.8	2.5 ± 3.2	0.298	1.6 ± 1.9	2.4 ± 2.9	0.357	1.9 ± 2.0	2.0 ± 2.8	0.972	2.1 ± 2.0	1.9 ± 2.7	0.359
**In lymphocytes (%)**												
CD3^+^ T cells	60.1 ± 22.4	62.2 ± 7.3	0.064	59.5 ± 24.0	62.5 ± 6.9	0.016^*^	61.3 ± 26.5	60.6 ± 7.7	0.258	61.4 ± 27.5	60.6 ± 7.5	0.887
γδ-T cells	4.8 ± 2.0	8.4 ± 7.3	0.079	4.5 ± 2.1	8.0 ± 6.7	0.047^*^	4.7 ± 2.2	7.3 ± 6.3	0.095	4.9 ± 2.1	7.0 ± 6.2	0.224
NKG2D^+^ γδ-T cells	4.0 ± 2.1	8.0 ± 7.1	0.050	3.8 ± 2.1	7.6 ± 6.5	0.025^*^	4.0 ± 2.1	6.7 ± 6.2	0.067	4.2 ± 2.1	6.5 ± 6.1	0.166
CD158a^+^ γδ-T cells	0.8 ± 0.5	1.4 ± 1.6	0.164	0.8 ± 0.5	1.3 ± 1.5	0.197	0.8 ± 0.5	1.2 ± 1.3	0.199	0.8 ± 0.5	1.2 ± 1.3	0.211
CD158b^+^ γδ-T cells	1.1 ± 1.2	1.7 ± 2.1	0.240	1.0 ± 1.2	1.7 ± 1.9	0.190	1.2 ± 1.2	1.4 ± 1.8	0.641	1.2 ± 1.3	1.4 ± 1.8	0.739

* *P < 0.05*.

### Power Analysis of NKG2D^+^ γδ-T cells in Predicting Pregnancy Failure in uRIF Patients

**Figure 3:**
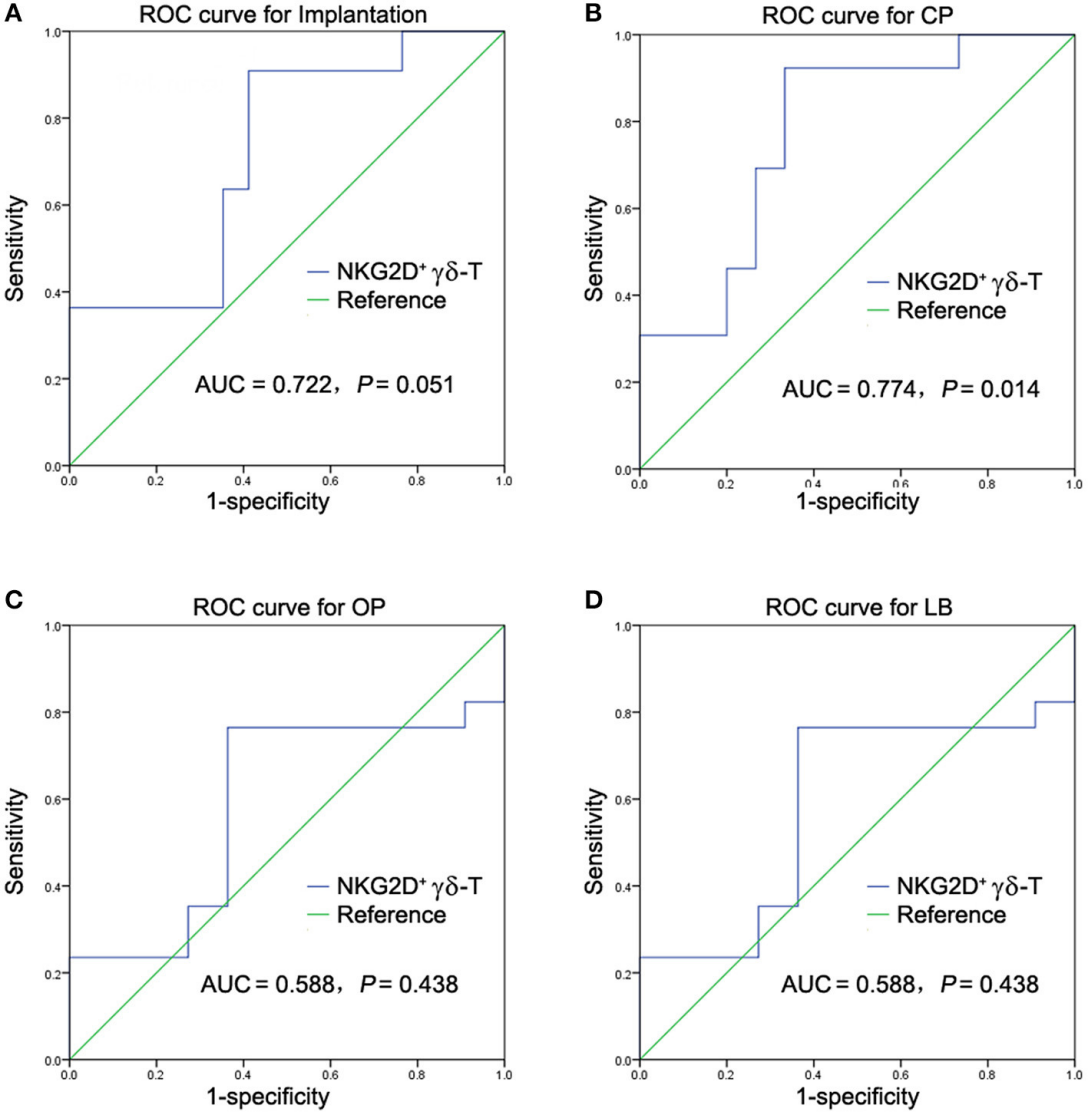
ROC analysis of NKG2D^+^ γδ-T cells in predicting pregnancy outcome failure in patients with uRIF. **(A)** Power analysis of NKG2D in predicting implantation outcome in patients with uRIF. **(B)** Power analysis of NKG2D in predicting clinical pregnancy outcome in patients with uRIF. **(C)** Power analysis of NKG2D in predicting ongoing pregnancy outcome in patients with uRIF. **(D)** Power analysis of NKG2D in predicting live birth outcome in patients with uRIF. AUC indicated the area of under curve of NKG2D^+^ γδ-T cells, and *P* indicated the statistical power of NKG2D^+^ γδ-T cells.

To further evaluate the predictive power and cut-off value of NKG2D^+^ γδ-T cells in predicting pregnancy failure in uRIF patients, 28 patients were randomly selected from the study population and analyzed by the ROC curve, while the remaining 10 patients were used to validate the power. The ROC curve showed that the accuracies of NKG2D^+^ γδ-T cells in predicting implantation, clinical pregnancy, ongoing pregnancy and live birth, measured by AUC, were 0.722 (95% CI: 0.526-0.917, *P* = 0.051, Figure 3A), 0.774 (95% CI: 0.597-0.951, *P* = 0.014, Figure 3B), 0.588 (95% CI: 0.366-0.811, *P* = 0.438, Figure 3C), and 0.588 (95% CI: 0.366-0.811, *P* = 0.438, Figure 3D), respectively. These results indicated that the frequency of NKG2D^+^ γδ-T cells in lymphocytes could be used as a potential indicator to predict clinical pregnancy failure in patients with uRIF. At a cut-off value of 3.24% referred to the percentage of NKG2D^+^ γδ-T cells in lymphocytes, the sensitivity and specificity were 92.3 and 66.7%, respectively. In the validation set, the sensitivity, specificity, positive predictive value, and negative predictive value of NKG2D^+^ γδ-T cells for clinical pregnancy failure were 80.0, 20.0, 50.0, and 50.0%, respectively. The high accuracy and sensitivity in predicting clinical pregnancy failure further indicated that the percentage of NKG2D^+^ γδ-T cells in lymphocytes was a potential prognostic indicator for clinical pregnancy failure in uRIF patients.

### Kaplan-Meier Survival Analysis Based on NKG2D^+^ γδ-T cells in Patients With uRIF

**Figure 4:**
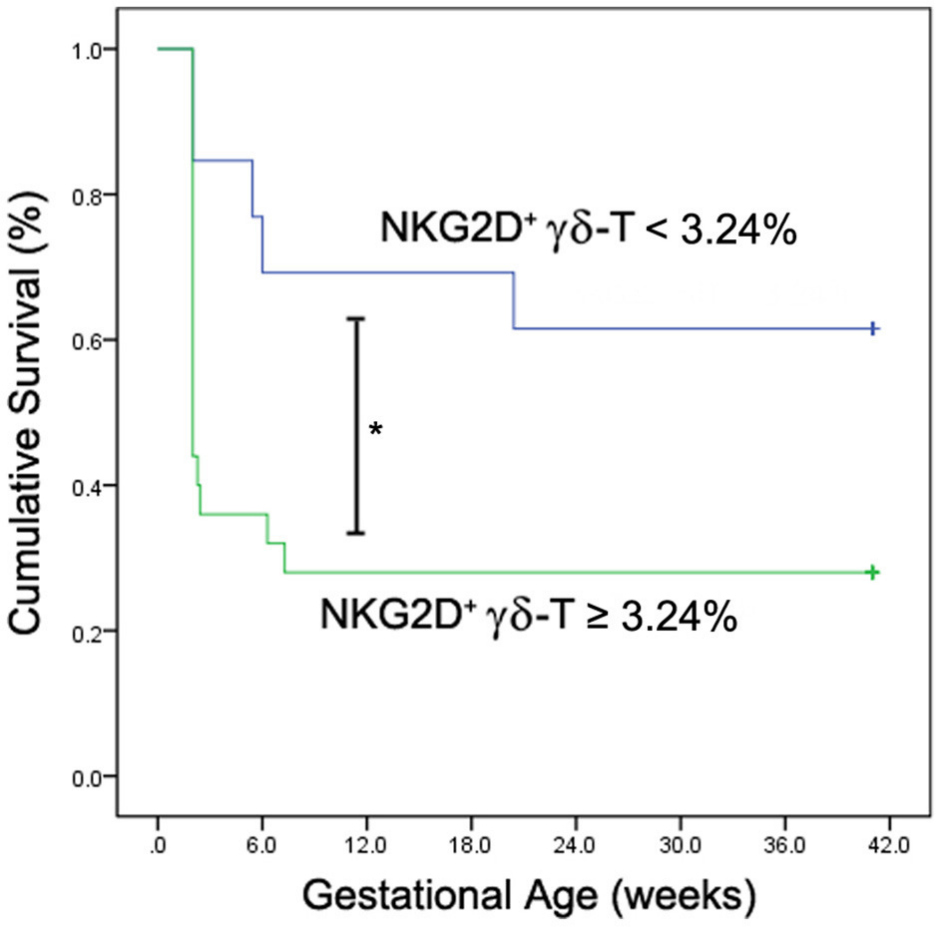
Kaplan-Meier survival analysis of NKG2D^+^ γδ-T cells for live birth rate in uRIF patients. The cumulative survival rate (live birth rate) was compared between uRIF patients with low frequency of NKG2D^+^ γδ-T cells (<3.24%, blue line) and those with high frequency of NKG2D^+^ γδ-T cells (≥3.24%, green line). *P < *0.05*.

The frequency of NKG2D^+^ γδ-T cells in lymphocytes allowed us to classify the uRIF patients into subgroups (group 1: NKG2D^+^ γδ-T cells≤3.24%; group 2: NKG2D^+^ γδ-T cells>3.24%; cut-off value was determined with the ROC curve). The clinical characteristics of these two groups are listed in Table 3, and the results showed no evident difference among the basic parameters in these two groups. The live birth rate of patients in the group 1 (NKG2D^+^ γδ-T cells <3.24%) and group 2 (NKG2D^+^ γδ-T cells ≥3.24%) were 61.5% and 28.0%, respectively. Kaplan-Meier survival curve (Figure 4) and the test of the equality of survival distribution (Supplementary Table 1) further demonstrated that the frequency of NKG2D^+^ γδ-T cells in lymphocytes negatively correlated with live birth rate in patients with uRIF. In addition, the estimated mean gestational age was 28 weeks in the group 1, while it was only 13 weeks in the group 2, suggesting that the presence of a low frequency of NKG2D^+^ γδ-T cells in lymphocytes corresponded to a significantly increased chance of successful live birth (Supplementary Table 2). Taken together, these data suggested that the frequency of NKG2D^+^ γδ-T cells in lymphocytes was negatively associated with the live birth rate and gestational age in uRIF patients.

**Table 3:** Clinical characteristics of uRIF patients categorized based on the cut-off value of percentage of NKG2D^+^ γδ-T cells in lymphocytes.

**Factors**	**NKG2D**^****+****^ ****γδ**-T cells**		***P***
	**Low group (*n* = 13)**	**High group (*n* = 25)**	
Age (years)	35.6 ± 3.9	35.1 ± 4.0	0.718
BMI (kg/m^2^)	22.3 ± 1.6	21.0 ± 2.3	0.083
No. of previous pregnancy failure	4.8 ± 2.6	4.5 ± 2.0	0.702
E2 (pg/mL)	43.3 ± 14.3	38.9 ± 16.2	0.414
P (ng/mL)	0.3 ± 0.1	0.3 ± 0.2	0.767
FSH (mIU/mL)	6.5 ± 1.8	6.9 ± 1.8	0.541
LH (mIU/mL)	4.9 ± 2.1	4.5 ± 1.6	0.546
PRL (ng/mL)	20.4 ± 7.0	18.2 ± 4.1	0.225
T (ng/mL)	0.5 ± 0.3	1.0 ± 1.6	0.286
Blastocyst/transferred embryos	61.5%	36.0%	0.178
No. of transferred embryos	1.9 ± 0.6	2.0 ± 0.6	0.585
No. of high-quality embryos	1.4 ± 0.8	1.6 ± 0.9	0.555

*BMI, body mass index; E2, estradiol; P, progesterone; FSH, follicle-stimulating hormone; LH, luteinizing hormone; PRL, prolactin; T, testosterone*.

## Discussion

Our study demonstrated that the frequency of peripheral NKG2D^+^ γδ-T cells in lymphocytes was significantly increased in the uRIF patients with failure of implantation or clinical pregnancy compared to that in the uRIF patients with corresponding successful pregnancy. The ROC analysis for the diagnostic power of NKG2D^+^ γδ-T cells resulted in an AUC of 0.774, with 92.3% sensitivity and 66.7% specificity in differentiating uRIF patients with successful clinical pregnancy from those with clinical pregnancy failure. Moreover, K-M survival curve analysis showed that the frequency of NKG2D^+^ γδ-T cells was negatively associated with live birth rate and gestational age. These results suggest that the NKG2D on γδ-T cell is a potential biochemical predictor for pregnancy outcome in uRIF patients.

Previous studies showed that peripheral γδ-T cells may be involved in recurrent miscarriage through the increased pro-inflammatory factors such as interleukin (IL)-12, IL-17, and IFN-γ, and the decreased anti-inflammatory factors such as IL-10 (17, 30). The increased NKG2C and decreased NKG2A on peripheral γδ-T cells were also reported to be associated with premature birth (31) and pre-eclampsia (32). In current study, we showed that the percentage of NKG2D^+^ γδ-T cells in lymphocytes was significantly increased in the uRIF patients with a failure in implantation or clinical pregnancy, when compared to that in the uRIF patients with successful implantation or successful clinical pregnancy. Taken together, these data indicated that abnormal expression of molecules regulating cytotoxicity of γδ-T cells was associated with adverse pregnancy outcomes. However, these studies were mainly based on the comparisons of the γδ-T cell status between women with adverse pregnancy and normal pregnancy, the prognosis value of these markers in reproductive failure was not investigated. Therefore, the clinical significance of these conclusions is greatly reduced. In this study, we demonstrated that the AUC of the frequency of NKG2D^+^ γδ-T cells in lymphocytes to predict the clinical pregnancy failure was 0.774, and the optimal cut-off value was 3.24% with 92.3% sensitivity and 66.7% specificity. Moreover, further K-M survival analysis showed that the lower live birth rate and shorter gestational age were observed in uRIF patients with a high frequency of NKG2D^+^ γδ-T cells. Our results indicate that NKG2D on γδ-T cell is a potential predictor for pregnancy outcome in uRIF patients. It is known that the cytotoxic activity of γδ-T cells against virus or cancer cells was determined by the expression of NKG2D and granzyme B (GrB) (22, 33–35). Recently we also found that the percentage of GrB^+^ γδ-T cells in lymphocytes was negatively associated with successful clinical pregnancy (36). These data suggest that the increase of γδ-T cell cytotoxicity is deleterious to the pregnancy.

The limitations of this study are reflected in the fact that, although the sensitivity of NKG2D^+^ γδ-T cells in predicting clinical pregnancy failure was as high as 80.0% during the validation phase, the specific, positive, and negative predictive values were as low as 20.0, 50.0, and 50.0%, respectively. The following three factors may contribute to them. First, the relatively small sample size in this study. The larger sample size, the greater statistical power and accuracy of the prediction. Therefore, a study with a larger sample size should be further performed to confirm this conclusion. Second, other receptors related to the cytotoxicity of γδ-T cells, such as NKG2C (31) and NKG2A (32), should be investigated in further study. Third, the appropriate interaction between receptors on immune cells and corresponding ligands on trophoblasts should be also considered because they are also associated with a successful pregnancy (37). The role of NKG2D at the maternal-fetal interface might be modified by the corresponding ligands expressed on trophoblasts. Previous study reported that the KIR genotype and haplotype is associated with pregnancy loss during IVF cycles. KIR B haplotype carriers experienced more pregnancy failure than KIR A haplotype carriers after embryo transfer. However, this risk was significantly influenced by their corresponding ligand, such as HLA-C alleles, present in the embryo. The high-risk combinations (KIR B/HLA-C1) resulted in a 51% increased risk of pregnancy failure (38). Therefore, the overall prognostic value of combined indicator with NKG2D and potential ligands on trophoblasts in predicting pregnancy outcomes in uRIF patients should be also further investigated.

Taken together, our study suggests that the increased frequency of peripheral NKG2D^+^ γδ-T cells in lymphocyte is significantly associated with pregnancy failure in uRIF patients. The ROC and K-M survival analysis indicate that NKG2D on γδ-T cell is a potential biomarker in predicting pregnancy outcome in uRIF patients. It is worth noting that finding effective treatment and evaluating their efficacy against NKG2D on γδ-T cells is a necessary step before this predictor can be applied in clinical translation.

## Data Availability Statement

The raw data supporting the conclusions of this article will be made available by the authors, without undue reservation.

## Ethics Statement

The studies involving human participants were reviewed and approved by Ethics Committee of Shenzhen Zhongshan Urology Hospital, Shenzhen, Guangdong Province, PR China. The patients/participants provided their written informed consent to participate in this study.

## Author Contributions

WT and CH designed the study, performed the data analysis, and wrote the manuscript. ZX performed the data analysis and critically reviewed the manuscript. YZh and YL performed the experiments and assisted in the preparation of the figures and tables. JX and HZ enrolled the subjects and collected the peripheral blood. YZe contributed to the study design, and critically reviewed the manuscript. All authors contributed to the article and approved the submitted version.

## Conflict of Interest

The authors declare that the research was conducted in the absence of any commercial or financial relationships that could be construed as a potential conflict of interest.

## References

[1] Datta J Palmer MJ Tanton C Gibson LJ Jones KG Macdowall W. Prevalence of infertility and help seeking among 15 000 women and men. Hum Reprod. (2016) 31:2108–18. 10.1093/humrep/dew123 27365525 PMC4991655

[2] Busnelli A Reschini M Cardellicchio L Vegetti W Somigliana E Vercellini P. How common is real repeated implantation failure? An indirect estimate of the prevalence. Reprod Biomed. (2019) 40:91–7. 10.1016/j.rbmo.2019.10.014 31924493

[3] Kolibianakis EM Venetis CA. Recurrent Implantation Failure. Boca Raton, FL: CRC Press (2019). 10.1201/9781315165707

[4] Huang C Zeng Y Tu W. The role of gammadelta-T cells during human pregnancy. Am J Reprod Immun. (2017) 78:2. 10.1111/aji.12713 28653491

[5] Huppertz B. The feto-maternal interface: setting the stage for potential immune interactions. Semin Immunopathol. (2007) 29:83–94. 10.1007/s00281-007-0070-7 17621696

[6] Chien YH Konigshofer Y. Antigen recognition by gammadelta T cells. Immunol Rev. (2007) 215:46–58. 10.1111/j.1600-065X.2006.00470.x 17291278

[7] Hayday AC. Gammadelta T cells and the lymphoid stress-surveillance response. Immunity. (2009) 31:184–96. 10.1016/j.immuni.2009.08.006 19699170

[8] Born WK Reardon CL O'Brien RL. The function of gammadelta T cells in innate immunity. Curr Opin Immunol. (2006) 18:31–8. 10.1016/j.coi.2005.11.007 16337364

[9] Bonneville M O'Brien RL Born WK. Gammadelta T cell effector functions: a blend of innate programming and acquired plasticity. Nat Rev Immun. (2010) 10:467–78. 10.1038/nri2781 20539306

[10] Zheng J Liu Y Lau YL Tu W. gammadelta-T cells: an unpolished sword in human anti-infection immunity. Cell Mol Immunol. (2013) 10:50–7. 10.1038/cmi.2012.43 23064104 PMC4003172

[11] Li J Li H Mao H Yu M Feng T Yang F. Vgamma9Vdelta2-T lymphocytes have impaired antiviral function in small-for-gestational-age and preterm neonates. Cell Mol Immunol. (2013) 10:253–60. 10.1038/cmi.2012.78 23524656 PMC4012779

[12] Chen Q Wen K Lv A Liu M Ni K Xiang Z. Human Vgamma9Vdelta2-T cells synergize CD4(+) T follicular helper cells to produce influenza virus-specific antibody. Front Immunol. (2018) 9:599. 10.3389/fimmu.2018.00599 29670614 PMC5893649

[13] Pei Y Wen K Xiang Z Huang C Wang X Mu X. CD137 costimulation enhances the antiviral activity of Vgamma9Vdelta2-T cells against influenza virus. Signal Transduct Target Ther. (2020) 5:74. 10.1038/s41392-020-0174-2 32488072 PMC7266814

[14] Polgar B Barakonyi A Xynos I Szekeres-Bartho J. The role of gamma/delta T cell receptor positive cells in pregnancy. Am J Reprod Immunol. (1999) 41:239–44. 10.1111/j.1600-0897.1999.tb00433.x 10374699

[15] Psarra K Kapsimali V Tarassi K Dendrinos S Athanasiadis T Botsis D. TCRgammadelta + T lymphocytes in unexplained recurrent spontaneous abortions. Am J Reprod Immunol. (2001) 45:6–11. 10.1111/j.8755-8920.2001.450102.x 11211948

[16] Barakonyi A Kovacs KT Miko E Szereday L Varga P Szekeres-Bartho J. Recognition of nonclassical HLA class I antigens by gamma delta T cells during pregnancy. J Immunol. (2002) 168:2683–8. 10.4049/jimmunol.168.6.2683 11884433

[17] Barakonyi A Polgar B Szekeres-Bartho J. The role of gamma/delta T-cell receptor-positive cells in pregnancy: part II. Am J Reprod Immunol. (1999) 42:83–7. 10476689

[18] Todaro M D'Asaro M Caccamo N Iovino F Francipane MG Meraviglia S. Efficient killing of human colon cancer stem cells by gammadelta T lymphocytes. J Immunol. (2009) 182:7287–96. 10.4049/jimmunol.0804288 19454726

[19] Costa G Loizon S Guenot M Mocan I Halary F de Saint-Basile G. Control of Plasmodium falciparum erythrocytic cycle: gammadelta T cells target the red blood cell-invasive merozoites. Blood. (2011) 118:6952–62. 10.1182/blood-2011-08-376111 22045985

[20] Xiang Z Tu W. Dual face of Vgamma9Vdelta2-T cells in tumor immunology: anti- versus pro-tumoral activities. Front Immunol. (2017) 8:1041. 10.3389/fimmu.2017.01041 28894450 PMC5581348

[21] Nedellec S Sabourin C Bonneville M Scotet E. NKG2D costimulates human V gamma 9V delta 2 T cell antitumor cytotoxicity through protein kinase C theta-dependent modulation of early TCR-induced calcium and transduction signals. J Immunol. (2010) 185:55–63. 10.4049/jimmunol.1000373 20511557

[22] Xiang Z Liu Y Zheng J Liu M Lv A Gao Y. Targeted activation of human Vgamma9Vdelta2-T cells controls epstein-barr virus-induced B cell lymphoproliferative disease. Cancer Cell. (2014) 26:565–76. 10.1016/j.ccr.2014.07.026 25220446

[23] Li H Xiang Z Feng T Li J Liu Y Fan Y. Human Vgamma9Vdelta2-T cells efficiently kill influenza virus-infected lung alveolar epithelial cells. Cell Mol Immunol. (2013) 10:159–64. 10.1038/cmi.2012.70 23353835 PMC4003054

[24] Qin G Liu Y Zheng J Ng IH Xiang Z Lam KT. Type 1 responses of human Vgamma9Vdelta2 T cells to influenza A viruses. J Virol. (2011) 85:10109–16. 10.1128/JVI.05341-11 21752902 PMC3196408

[25] Qin G Liu Y Zheng J Xiang Z Ng IH Malik Peiris JS. Phenotypic and functional characterization of human gammadelta T-cell subsets in response to influenza A viruses. J Infect Dis. (2012) 205:1646–53. 10.1093/infdis/jis253 22457284

[26] Lesport E Baudhuin J Sousa S LeMaoult J Zamborlini A Rouas-Freiss N. Inhibition of human gamma delta [corrected] T-cell antitumoral activity through HLA-G: implications for immunotherapy of cancer. Cell Mol Life Sci. (2011) 68:3385–99. 10.1007/s00018-011-0632-7 21337044 PMC11114898

[27] Biassoni R Cantoni C Falco M Verdiani S Bottino C Vitale M. The human leukocyte antigen (HLA)-C-specific “activatory” or “inhibitory” natural killer cell receptors display highly homologous extracellular domains but differ in their transmembrane and intracytoplasmic portions. J Exp Med. (1996) 183:645–50. 10.1084/jem.183.2.645 8627176 PMC2192451

[28] Niu C Li M Zhu S Chen Y Zhou L Xu D. Decitabine inhibits gamma delta T cell cytotoxicity by promoting KIR2DL2/3 expression. Front Immunol. (2018) 9:617. 10.3389/fimmu.2018.00617 29632540 PMC5879086

[29] Dolstra H Fredrix H van der Meer A de Witte T Figdor C van de Wiel-van Kemenade E. TCR gamma delta cytotoxic T lymphocytes expressing the killer cell-inhibitory receptor p58.2 (CD158b) selectively lyse acute myeloid leukemia cells. Bone Marrow Trans. (2001) 27:1087–93. 10.1038/sj.bmt.1703043 11438826

[30] Talukdar A Rai R Aparna Sharma K Rao DN Sharma A. Peripheral Gamma Delta T cells secrete inflammatory cytokines in women with idiopathic recurrent pregnancy loss. Cytokine. (2018) 102:117–22. 10.1016/j.cyto.2017.07.018 28802663

[31] Szereday L Barakonyi A Miko E Varga P Szekeres-Bartho J. Gamma/deltaT-cell subsets, NKG2A expression and apoptosis of Vdelta2+ T cells in pregnant women with or without risk of premature pregnancy termination. Am J Reprod Immunol. (2003) 50:490–6. 10.1046/j.8755-8920.2003.00107.x 14750557

[32] Miko E Szereday L Barakonyi A Jarkovich A Varga P Szekeres-Bartho J. Immunoactivation in preeclampsia: Vdelta2+ and regulatory T cells during the inflammatory stage of disease. J Reprod Immunol. (2009) 80:100–8. 10.1016/j.jri.2009.01.003 19395088

[33] Qin G Mao H Zheng J Sia SF Liu Y Chan PL. Phosphoantigen-expanded human gammadelta T cells display potent cytotoxicity against monocyte-derived macrophages infected with human and avian influenza viruses. J Infect Dis. (2009) 200:858–65. 10.1086/605413 19656068 PMC7110194

[34] Tu W Zheng J Liu Y Sia SF Liu M Qin G. The aminobisphosphonate pamidronate controls influenza pathogenesis by expanding a gammadelta T cell population in humanized mice. J Exp Med. (2011) 208:1511–22. 10.1084/jem.20110226 21708931 PMC3135369

[35] Wang X Xiang Z Liu Y Huang C Pei Y Wang X. Exosomes derived from Vdelta2-T cells control Epstein-Barr virus-associated tumors and induce T cell antitumor immunity. Sci Transl Med. (2020) 12:563. 10.1126/scitranslmed.aaz3426 32998970

[36] Huang C Zhang Y Xiang Z Li Y Lin R Xu J. Granzyme B-expressing γδ-T and NK cells as a predictor of clinical pregnancy failure in patients with unexplained repeated implantation failure. J Reprod Immunol. (2020) 2020:103269. 10.1016/j.jri.2020.103269 33540297

[37] Rabot M Tabiasco J Polgar B Aguerre-Girr M Berrebi A Bensussan A. HLA class I/NK cell receptor interaction in early human decidua basalis: possible functional consequences. Chem Immunol Allerg. (2005) 89:72–83. 10.1159/000087914 16129954

[38] Morin SJ Treff NR Tao X Scott RT 3rd Franasiak JM Juneau CR. Combination of uterine natural killer cell immunoglobulin receptor haplotype and trophoblastic HLA-C ligand influences the risk of pregnancy loss: a retrospective cohort analysis of direct embryo genotyping data from euploid transfers. Fert Ster. (2017) 107:677–83 e2. 10.1016/j.fertnstert.2016.12.004 28069185

