# Supplementary material for: NKG2D as a Cell Surface Marker on γδ-T Cells for Predicting Pregnancy Outcomes in Patients With Unexplained Repeated Implantation Failure
Source: Front Immunol. 2021 Mar 10;12:631077. doi: 10.3389/fimmu.2021.631077 (PMC7988228; doi:10.3389/fimmu.2021.631077)
Supplement: Supplementary file 1 [file Table_1.docx]

**Supplementary Table 1. Kaplan-Meier test of NKG2D^+^ γδ-T cells for live birth rate in uRIF patients.**

| **Test of equality** | | **Chi-square** | **Df** | | **Significance** | | |
| --- | --- | --- | --- | --- | --- | --- | --- |
| NKG2D^+^ γδ-T | |  |  | |  | | |
| Log Rank (Mantel-Cox) | 4.469 | | | 1 | | 0.035 |  |
| Breslow (Generalized Wilcoxon) | 5.797 | | | 1 | | 0.016 |  |
| Tarone-Ware | 5.302 | | | 1 | | 0.021 |  |
