# Supplementary material for: NKG2D as a Cell Surface Marker on γδ-T Cells for Predicting Pregnancy Outcomes in Patients With Unexplained Repeated Implantation Failure
Source: Front Immunol. 2021 Mar 10;12:631077. doi: 10.3389/fimmu.2021.631077 (PMC7988228; doi:10.3389/fimmu.2021.631077)
Supplement: Supplementary file 2 [file Table_2.docx]

**Supplementary Table 2.** **Means of gestational age of patients categorized according to the percentage of NKG2D^+^ γδ-T cells**

| **parameters** | **Mean** | | | |
| --- | --- | --- | --- | --- |
|  | **Estimate** | **Std. Error** | **95% Confidence Interval** | |
|  |  |  | **Lower Bound** | **Upper Bound** |
| NKG2D^+^ γδ-T |  |  |  |  |
| < 3.24% | 27.989 | 4.714 | 18.750 | 37.228 |
| ≥3.24% | 13.331 | 3.460 | 6.549 | 20.114 |
| Overall | 18.346 | 3.009 | 12.448 | 24.244 |
